# A retrospective cross-sectional survey on nosocomial bacterial infections and their antimicrobial susceptibility patterns in hospitalized patients in northwest of Iran

**DOI:** 10.1186/s13104-021-05503-0

**Published:** 2021-03-09

**Authors:** Hamid Sadeghi, Saeideh Gholamzadeh Khoei, Mehdi Bakht, Mohammad Rostamani, Sara Rahimi, Mehdi Ghaemi, Bahman Mirzaei

**Affiliations:** 1 Department of Microbiology and Virology, School of Medicine, Zanjan University of Medical Sciences, Zanjan, Iran; 2 Molecular Medicine Research Center, Hamadan University of Medical Sciences, Hamadan, Iran; 3 Department of Microbiology, Qazvin University of Medical Sciences, Qazvin, Iran; 4 Department of Anesthesiology, School of Medicine, Zanjan University of Medical Sciences, Zanjan, Iran; 5 Department of Microbiology and Virology, School of Medicine, Mazandaran University of Medical Sciences, Mazandaran, Iran

**Keywords:** Nosocomial infections, Bacterial isolates, Susceptibility patterns, Multi-drug resistant (MDR), Extensively-drug resistant (XDR)

## Abstract

**Objective:**

Nosocomial infections (NIs) are known as one of the remarkable problems in all countries. This study is aimed to estimate the prevalence rate of nosocomial bacterial agents with antimicrobial susceptibility pattern in hospitalized patients. This study was conducted from April 2017 to September 2018, on 4029 hospitalized patients. We set out to recognize the commonest bacterial infections and antimicrobial susceptibility patterns of nosocomial infection.

**Results:**

Of the 4029 patients, 509 (12.6%) of them were culture positive. Of these *Escherichia coli* (*E. coli*) (98.3%) and *Staphylococcus epidermidis* (*S. epidermidis*) (37.5%) were the most abundant bacterial identified in the urinary tract and bloodstream cultures respectively, Moreover, *Acinetobacter spp.* (100%) and *Pseudomonas aeruginosa* (22.2%) were the most abundant organisms detected in the respiratory system. According to susceptibility testing results, 370 (80.5%) and 264 (57.3%) in Gram-negatives and 44 (91.7%) and 35 (72.9%) in gram positives isolated strains were classified as multidrug-resistant (MDR) and extensive drug-resistant (XDR) strain respectively. On account of the high prevalence of MDR and XDR bacterial species, there is a pressing need for the expansion of new strategies on antibiotic supervision and infection control to introduce new guideline on empirical antibiotic therapy.

**Supplementary Information:**

The online version contains supplementary material available at 10.1186/s13104-021-05503-0.

## Introduction

Nosocomial infections (NIs) are known as hospital-gained infection expanding within 48–72 h after incoming [1]. A large part of morbidity and mortality in hospitals goes back to the NIs [2]. Following the increasing rate of NIs, developing in socio-economic disturbance, antimicrobial resistance, and the mortality rate are inevitable [3]. NIs happen throughout the world both in developing and developed countries. NIs accounts for 10% in developing and 7% in developed countries [4]. Nearly 2 million people have been tangled in this matter and also would be known as a major reason for the loss of life and money [5]. Bacteria are hugely the prominent cause of NIs [6]. The most usual kinds of NIs which happen in a hospital set up are: urinary system, bloodstream infections, surgical site infections, gastroenteritis, respiratory system [7, 8]. Despite global endeavor to rein NIs during previous years, NIs still stays a widespread problem and as one of the important causes of antibiotic resistance in hospitalized patients [9]. As antibiotic resistance continues to greaten, attuned definitions with which to characterize and recognize multiple resistant bacteria to antimicrobial agents are indispensable; therefor epidemiological information can be trustworthily gathered and compared among healthcare settings and countries [10–13]. In verbal terms, multidrug-resistant (MDR) means ‘resistant to more than one antimicrobial agent’, but no standardized descriptions for MDR have been come to an agreement upon yet by the medical community [14, 15]. Extensive drug-resistant (XDR) Bacteria are epidemiologically remarkable in two reasons: resistance to multiple antimicrobial agents and resistance to all, or almost all, approved antimicrobial agents [16, 17]. In another definition MDR means non-susceptibility to at minim one agent in three or more antimicrobial categories, XDR means non-susceptibility to at minim one agent in all but two or fewer antimicrobial categories [18]. In bacteria, integrons as transposable elements and extended spectrum β-lactamases (ESBLs) enzymes in a variety of Gram negative bacteria are related to an increased resistance to commonly used antibiotics [19–21]. There is no denying the fact that if there is no efficient timely respond, the problem of resistance to antibiotic is becoming a huge difficulty in the future years. Therefore, the aims of this study were to estimate the prevalence rate of nosocomial bacterial agents with antimicrobial susceptibility patterns in hospitalized patients referred to Emdadi Hospital, Abhar, Iran.

## Material and methods

### Study area, study period and study population

This retrospective cross-sectional study was conducted on 4029 patients from April 2017 to September 2018 in the Emdadi Hospital, Abhar, Iran. NIs criteria were matching to the Center for Disease Control and Prevention (CDC) definitions [22].

### Identification of bacterial isolates

After hospitalizing, hospitalized patients would be followed-up in order to identify the cause of the infection, which requires sampling and transferring them to laboratory. In the continuation of this process, after diagnosing the cause of the infection, it is the duty of the laboratory to determine the antibiotic resistance pattern to physician so that prescribe the most appropriate and effective antibiotic for treatment [23]. Samples including morning midstream urine, blood, stool, wound discharge, and respiratory samples were aseptically gathered via sterile containers and carried immediately to the Microbiology laboratory with proper transport media. Identity of bacteria was done via colony specifications, gram reaction, and divers biochemical examinations following standard methods [24]. Briefly, samples were grown on selective media including blood agar, MacConkey agar, and eosin methylene blue (EMB) medium. Then, bacterial identification of the isolates was performed according to the previously published [24].

### Antibiotics susceptibility test

Confirmed isolates by biochemical tests underwent the disc diffusion susceptibility test based on the clinical and laboratory standards institute guidelines (CLSI) [25]. In short, a 0.5 McFarland suspension of each isolate was inoculated on a whole plate surface Mueller–Hinton agar (Pronadisa, Spain) plate by streaking the swab in back and forth motions. Antimicrobial impregnated discs (Padtanteb, Iran) including co-trimoxazole (1.25/23.75 μg), gentamycin (10 μg), amikacin (30 μg), ciprofloxacin (5 μg), and erythromycin (15 μg) to Gram positive isolates and ceftriaxone (30 μg), ciprofloxacin (5 μg), gentamycin (10 μg), nalidixic acid (30 μg), and nitrofurantoin (300 μg) and co-trimoxazole (1.25/23.75 μg) to Gram-negative isolates were put on the surface of the agar, and the plates were incubated for 24 h at 37°C. Following incubation, inhibition zone sizes to the nearest millimeter were measured using a ruler. Using published CLSI guidelines, susceptibility, or resistance of the organism to each tested drug was determined. Interpretation of antibiotic susceptibility to evaluated MDR and XDR isolates was performed based on the European center for disease prevention and control( ECDC) instructor as well [18]. *E. coli* ATCC 25922 and *S. epidermidis* ATCC 12228 were served as positive controls in all phenotypic procedures as well.

### Data analysis and interpretation

Data were analyzed using SPSS 16.0 and results were presented through tables. This study was designed to estimate the prevalence rate of nosocomial bacterial agents with antimicrobial susceptibility patterns and determining the rate of XDR and MDR in isolated bacteria from Inpatient, thus we did not consider demographic information of patients.

## Result

During 1-year study, 4029 samples with nosocomial infections were collected. Out of 509 obtained isolates, 481 (94.49%) were gathered from urine, followed by 12 (2.35%) respiratory system, 10 (1.96%) blood, 5 (0.98%) wound, and 1 (0.19%) stool. Detailed data listed in Additional file 1, Additional file 2. The most common pathogens recognized in the urinary tract and bloodstream cultures were *E. coli* (98.3%) and *S. epidermidis* (37.5%) respectively, In addition, *Acinetobacter spp.* (100%) and *P. aeruginosa* (22.2%) were the most common organisms isolated from the respiratory system. Details of the prevalence of isolated bacteria from different samples are shown in Table 1. Antibiotic resistance patterns of Gram-negative bacteria showed that the highest resistance rate was against nalidixic acid (67.2%) followed by co-trimoxazole (63.8%), and the lowest rate, 17.8%, was related to gentamicin respectively (Detailed data was presented in Table 2). *P. aeruginosa* and *Acinetobacter spp*. showed the most resistance to co-trimoxazole, ciprofloxacin, nalidixic acid, and nitrofurantoin. In addition, *Klebsiella spp.* displayed the highest resistance to ceftriaxone. Among Gram-positives, the greatest resistance was shown in erythromycin and co-trimoxazole with 89.6% and 81.2%% respectively. On the other side, in Table 1, the lowermost rates were linked to amikacin (27.1%) and gentamicin (25.0%) respectively. In this, *Enterococcus faecalis* (*E. faecalis*) showed 100% resistance to co-trimoxazole. Beyond that, *E. faecalis* was approximately resistant to all selected antibiotics except amikacin. Among Gram-negatives base on susceptibility testing results, 370 (80.5%) and 264 (57.3%) isolated strains were categorized as MDR and XDR strain respectively. Among Gram-positives according to susceptibility testing results, 44 (91.7%) and 35 (72.9%) isolated strains were also classified as MDR and XDR strain respectively. (Data are presented in Additional file 3, Additional file 4). According to our study design, the frequencies of nosocomial bacterial agents, antimicrobial susceptibility patterns and the rate of XDR and MDR in Inpatient were the priority of the current study. Therefore investigating any correlations with patient characteristics were out of this study goals.

**Table 1:** Isolation rates of bacteria considering clinical specimens (n%)

Bacterial isolates	Urine	Blood	Wound	Respiratory	Stool	Total
*E. coli*	354 (98.3%)	5 (1.38%)	1 (0.2%)	0 (0%)	0 (0%)	360 (100%)
*Citribacter spp*	25 (100%)	0 (0%)	0 (0%)	0 (0%)	0 (0%)	25 (100%)
*Klebsiella spp*	34 (77.2%)	1 (2.27%)	2 (4.54%)	7 (15.9%)	0 (0%)	44 (100%)
*Enterobacter spp*	16 (100%)	0 (0%)	0 (0%)	0 (0%)	0 (0%)	16 (100%)
*Serratia*	1 (100%)	0 (0%)	0 (0%)	0 (0%)	0 (0%)	1 (100%)
*Proteus spp*	2 (100%)	0 (0%)	0 (0%)	0 (0%)	0 (0%)	2 (100%)
*P. aeruginosa*	7 (77.8%)	0 (0%)	0 (0%)	2 (22.2%)	0 (0%)	9 (100%)
*Acinetobacter*	0 (0%)	0 (0%)	0 (0%)	3 (100%)	0 (0%)	3 (100%)
*Shigella sonnei*	0 (0%)	0 (0%)	0 (0%)	0 (0%)	1 (100%)	1 (100%)
*S. aureus*	20 (86.9%)	1 (4.34%)	2 (8.69%)	0 (0%)	0 (0%)	23 (100%)
*S. saprophyticus*	10 (100%)	0 (0%)	0 (0%)	0 (0%)	0 (0%)	10 (100%)
*S. epidermidis*	5 (62.5%)	3 (37.5%)	0 (0%)	0 (0%)	0 (0%)	8 (100%)
*E. faecalis*	4 (100%)	0 (0%)	0 (0%)	0 (0%)	0 (0%)	4 (100%)
*S. agalactiae*	3 (100%)	0 (0%)	0 (0%)	0 (0%)	0 (0%)	3 (100%)
Total	481 (94.49%)	10 (1.96%)	5 (0.98%)	12 (2.35%)	1 (0.19%)	509 (100.0%)

**Table 2:** Antibiotic susceptibility testing of Isolated strains

(n%) Antibiotic susceptibility patterns											
Bacterial isolates			SXT	GM	AN	NA	CRO	CP	FM	CN	E
G +	*S. aureus*	S	3 (13.0%)	10 (43.5%)	16 (69.6%)	ND	ND	10 (43.5%)	ND	2 (8.7%)	0 (0%)
		I	1 (4.3%)	9 (39.1%)	3 (13.0%)	ND	ND	3 (13.0%)	ND	6 (26.1%)	1 (4.3%)
		R	19 (82.6%)	4 (17.4%)	4 (17.4%)	ND	ND	10 (43.5%)	ND	15 (65.2%)	22 (95.7%)
	*S. saprophyticus*	S	3 (30.0%)	3 (30.0%)	9 (90.0%)	ND	ND	4 (40.0%)	ND	8 (80.0%)	2 (20.0%)
		I	1 (10.0%)	4 (40.0%)	0 (0%)	ND	ND	3 (30.0%)	ND	1 (10.0%)	1 (10.0%)
		R	6 (60.0%)	3 (30.0%)	1 (10.0%)	ND	ND	3 (30.0%)	ND	1 (10.0%)	7 (70.0%)
	*S. epidermidis*	S	1 (12.5%)	5 (62.5%)	3 (37.5%)	ND	ND	3 (37.5%)	ND	3 (37.5%)	0 (0%)
		I	0 (0%)	1 (12.5%)	0 (0%)	ND	ND	0 (0%)	ND	2 (25.0%)	0 (0%)
		R	7 (87.5%)	2 (25.0%)	5 (62.5%)	ND	ND	5 (62.5%)	ND	3 (37.5%)	8 (100.0%)
	*E. faecalis*	S	0 (0%)	1 (25.0%)	2 (50.0%)	ND	ND	0 (0%)	ND	0 (0%)	0 (0%)
		I	0 (0%)	0 (0%)	0 (0%)	ND	ND	0 (0%)	ND	1 (25.0%)	0 (0%)
		R	4 (100%)	3 (75.0%)	2 (50.0%)	ND	ND	4 (100.0%)	ND	3 (75.0)%	4 (100.0%)
	*E. faecalis*	S	0 (0%)	2 (66.7%)	2 (66.7%)	ND	ND	2 (66.7%)	ND	2 (66.7%)	1 (33.3%)
		I	0 (0%)	1 (33.3%)	0 (0%)	ND	ND	1 (33.3%)	ND	0 (0%)	0 (0%)
		R	3 (100.0%)	0 (0%)	1 (33.3%)	ND	ND	0 (0%)	ND	1 (33.3%)	2 (66.7%)
G-	*E. coli*	S	122 (33.9%)	101 (28.1%)	ND	88 (24.4%)	189 (52.5%)	224 (62.2%)	198 (55.0%)	ND	ND
		I	10 (2.8%)	195 (54.2%)	ND	45 (12.5%)	17 (4.7%)	16 (4.4%)	123 (34.2%)	ND	ND
		R	228 (63.3%)	64 (17.8%)	ND	227 (63.1%)	154 (42.8%)	120 (33.3%)	39 (10.8%)	ND	ND
	*Citribacter spp*	S	7 (28.0%)	11 (44.0%)	ND	0 (0%)	11 (44.0%)	16 (64.0%)	10 (40.0%)	ND	ND
		I	2 (8.0%)	10 (40.0%)	ND	2 (8.0%)	2 (8.0%)	2 (8.0%)	12 (48.0%)	ND	ND
		R	16 (64.0%)	4 (16.0%)	ND	23 (92.0%)	12 (48.0%)	7 (28.0%)	3 (12.0%)	ND	ND
	*Klebsiella spp*	S	12 (27.3%)	10 (22.7%)	ND	6 (13.6%)	15 (34.1%)	27 (61.4%)	15 (34.1%)	ND	ND
		I	4 (9.1%)	22 (50.0%)	ND	6 (13.6%)	1 (2.3%)	1 (2.3%)	6 (13.6%)	ND	ND
		R	28 (63.6%)	12 (27.3%)	ND	32 (72.7%)	28 (63.6%)	16 (36.4%)	23 (52.3%)	ND	ND
	*Enterobacter spp*	S	5 (31.2%)	5 (31.2%)	ND	1 (6.2%)	3 (18.8%)	9 (56.2%)	8 (50.0%)	ND	ND
		I	2 (12.5%)	3 (18.8%)	ND	2 (12.5%)	2 (12.5%)	0 (0%)	4 (25.0%)	ND	ND
		R	9 (56.2%)	8 (50.0%)	ND	13 (81.2%)	11 (68.8%)	7 (43.8%)	4 (25.0%)	ND	ND
	*Serratia*	S	1 (100.0%)	0 (0%)	ND	0 (0%)	1 (100.0%)	1 (100.0%)	0 (0%)	ND	ND
		I	0 (0%)	1 (100%)	ND	0 (0%)	0 (0%)	0 (0%)	1 (100.0%)	ND	ND
		R	0 (0%)	0 (0%)	ND	1 (100.0%)	0 (0%)	0 (0)%	0 (0%)	ND	ND
	*Proteus spp*	S	1 (50.0%)	1 (50.0%)	ND	0 (0%)	2 (100.0%)	2 (100.0%)	0 (0%)	ND	ND
		I	0 (0%)	1 (50.0%)	ND	1 (50%)	0 (0%)	0 (0%)	0 (0%)	ND	ND
		R	1 (50.0%)	0 (0%)	ND	1 (50%)	0 (0%)	0 (0%)	2 (100.0%)	ND	ND
	*P. aeruginosa*	S	0 (0%)	2 (22.2%)	ND	0 (0%)	2 (22.2%)	4 (44.4%)	0 (0%)	ND	ND
		I	0 (0%)	3 (33.3%)	ND	0 (0%)	2 (22.2%)	1 (11.1%)	0 (0%)	ND	ND
		R	9 (100%)	4 (44.4%)	ND	9 (100.0%)	5 (55.6%)	4 (44.4%)	9 (100.0%)	ND	ND
	*Acinetobacter*	S	0 (0%)	0 (0%)	ND	0 (0%)	0 (0%)	2 (66.7%)	1 (33.3%)	ND	ND
		I	0 (0%)	2 (66.7%)	ND	0 (0%)	0 (0%)	2 (66.7%)	0 (0%)	ND	ND
		R	3 (100%)	1 (33.3%)	ND	3 (100.0%)	3 (100.0%)	1 (33.1%)	2 (66.7%)	ND	ND
	*Shigella sonnei*	S	0 (0%)	1 (100.0%)	ND	0 (0%)	1 (100.0%)	0 (0%)	0 (0%)	ND	ND
		I	0 (0%)	0 (0%)	ND	0 (0%)	0 (0%)	0 (0%)	1 (100.0%)	ND	ND
		R	1 (100.0%)	0 (0%)	ND	1 (100.0%)	0 (0%)	1 (100.0%)	0 (0%)	ND	ND

*G+* Gram positive bacteria, *G−* gram negative bacteria, *S* sensitive, *I* intermediate, *R* resistant, *ND* not determined, *SXT* cotrimoxazole, *GM* gentamycin, *AN* amikacin, *NA* nalidixic acid, *CRO* ceftriaxone, *FM* nitrofurantoin, *CN* cephalexin, *E* erythromycin

## Discussion

Hospitals should have a pliable, naive, and regularly updated antibiotic-prescribing policy on a disease specific basis, relying whenever possible on knowledge of prevailing antibiotic-sensitivity patterns and controlled use of reserve antibiotics. This should incorporate local practice guidelines [23]. The microbiology laboratory has a major role in antimicrobial resistance decline that some of which are briefly mentioned: carrying out antibiotic susceptibility testing of appropriate microbial isolates consistent with standards, specifying which antibiotics are tested and reported for each organism, performing extra antibiotics testing for selected resistant bacteria, monitoring and reporting the prevalence of bacterial resistance to antimicrobial agents, limiting use of topical antibiotics and ensuring proper use of antibiotics (optimal choice, dosage and duration of antimicrobial therapy and chemoprophylaxis according to defined hospital antibiotic policy, and up-to-date antimicrobial guidelines) [23]. Organizational information about occupational infectious risks, exposures, and illnesses with occupational health services is regularly reviewed, updated, and are available in every hospital department. There must also be effective support at the national and regional levels [23]. Accordingly develop a system for identifying, investigating, reporting, analyzing, and controlling Healthcare-associated infection by giving some clues to a physician related to the suitable use of antibiotics and expanding antibiotic policies when antibiotic-resistant strains are detected could be essential [23]. Our study indicates a high prevalence (12.63%) of nosocomial infections in our hospital and in overall a high antibiotic resistance in the form of MDR and XDR (81.5%) among the pathogens. In the present research, the most frequencies of bacteria were *E. coli*, *Klebsiella spp*, *Citrobacter spp*, *S. aureus*, *Enterobacter spp*, *S. saprophyticus*, *P. aeruginosa, S. epidermidis, E. faecalis, Acinetobacter spp, S. agalactiae, Proteus spp, Serratia marcescens,* and *Shigella sonnei* respectively. In this study, the most common isolated bacteria were *E. coli* (70.7%), *Klebsiella spp* (8.6%), and *Citrobacter spp* (4.9%) and *S. aureus* (4.9%). other Iranian study conducted by Zahedi et al. was in line with our study [26]. On the other side, *P. aeruginosa* and *Acinetobacter spp* were detected as the most common bacteria by Davoudi et al. [27]. Another study performed in Iran, approved our study in this research, *Klebsiella spp*, *P. aeruginosa* and *E. coli* were more prevalent than other bacteria [28]. Farshid Rahimi-Bashar et al.performed a research in Iran and emphasized that *E. coli* is the most common strain followed by *Klebsiella spp* and this is in conformity with the results obtained in this study [29]. Study after study has shown that the UTI is the most frequent nosocomial infection in the world [30] and our study imply this subject and this is consistent with our study, because we also had the most nosocomial infections in UTI. As shown in Table 2, the nalidixic acid, co-trimoxazole were respectively the top two least effective antibiotics in the present study, and nitrofurantoin, gentamicin and amikacin were the lowest rate of resistance. Our study, similar to another Iranian research [31], showed the high efficiency of amikacin, and gentamycin for the treatment of nosocomial infections in Iran. Another study performed by Rajabi et al. was inconformity with the result of our work and the most prevalent bacteria was *Acinetobacter spp* and the most type of infection was respiratory system infections and he highest resistance rate was against ciprofloxacin [32]. Similar to our results, in the study of Zamani et al., gram-negative bacteria were the most frequent causes of nosocomial infections [33]. Similarly, in a survey performed on nosocomial UTI in a hospital, *E. coli* was the most isolated bacteria followed by *Klebsiella spp* [34]. These results were also shown in similar studies in Iran [35, 36] and other countries [37–39]. In contrast with our results, some studies in the United States [40] and Italy [41] reported *P. aeruginosa* and *S. aureus* as the most common pathogens. In 2012, and 2017some cross-sectional studies in Sudan and Iran have reported the percentage of MDR Enterobacteriaceae isolates spp., like 92.2%, and 74%. Frequencies of MDR isolated *E. coli* (66.6%), *K. pneumoniae* (95.8%), and Enterobacter *spp*., (80%) strain in 2015 were reported in a cross-sectional published study in Iran as well [42]. In our findings, MDR and XDR isolates were more assessed in Klebsiella sp., (93.1% and 79.5%) and *E. coli* (76.1% and 52.2%) too. Considering the sample size our finding shown similarities to mentioned studies in Iran and reconfirmed them [42].

As a result, high prevalence of MDR and XDR strains in the northwest of Iran regions is a serious issue in hospital wards. These findings insist on systematic effort to educate and persuade prescribers of antimicrobials to follow evidence-based prescribing, in order to stem antibiotic overuse, and thus antimicrobial resistance.

### Limitation

Responsible genes to antibiotic resistance, genetic relationship between the resistant strains, and investigating any correlations with patient characteristics are not determined and these are the limitation of this study. Moreover, identification of isolates merely performed taking advantage of biochemical aspect.

## Supplementary Information

**Additional file 1: **Frequency of positive and negative culture in clinical specienmence.

**Additional file 2: **Frequency and Percent of bacterial isolates recovered form hospitalized patients.

**Additional file 3: **Frequency of multi and extensively- drug resistant (MDR and XDR) Gram-negative isolated bacteria (n %)

**Additional file 4: ** Frequency of MDR and XDR Gram positive bacterial isolates (n %).

## Data Availability

All results of this study have been classified and maintained by a dissertation in the Zanjan University of medical Sciences. We have indeed provided all raw data on which our study is based. In addition, the datasets analysed during the current study available from the corresponding author on reasonable request.
