# Supplementary material for: A retrospective cross-sectional survey on nosocomial bacterial infections and their antimicrobial susceptibility patterns in hospitalized patients in northwest of Iran
Source: BMC Res Notes. 2021 Mar 9;14:88. doi: 10.1186/s13104-021-05503-0 (PMC7941966; doi:10.1186/s13104-021-05503-0)
Supplement: Supplementary file 1 — Additional file 1: Frequency of positive and negative culture in clinical specienmence. [file 13104_2021_5503_MOESM1_ESM.docx]

| Sample types | | Frequency | Percent |
| --- | --- | --- | --- |
| Urine | Negative samples | 3021 | 86.3% |
|  | Positive samples | 481 | 13.7% |
|  | Total | 3502 | 100% |
| Blood | Negative samples | 277 | 96.5% |
|  | Positive samples | 10 | 3.5% |
|  | Total | 287 | 100% |
| Wound | Negative samples | 11 | 68.8% |
|  | Positive samples | 5 | 31.2% |
|  | Total | 16 | 100% |
| Respiratory | Negative samples | 7 | 36.8% |
|  | Positive samples | 12 | 63.2% |
|  | Total | 19 | 100% |
| Stool | Negative samples | 204 | 99.5% |
|  | Positive samples | 1 | 0.5% |
|  | Total | 205 | 100% |
| Total all samples | | **4029** | |

Additional file 1: Frequency of positive and negative culture of clinical specienmence.
