# Supplementary material for: A retrospective cross-sectional survey on nosocomial bacterial infections and their antimicrobial susceptibility patterns in hospitalized patients in northwest of Iran
Source: BMC Res Notes. 2021 Mar 9;14:88. doi: 10.1186/s13104-021-05503-0 (PMC7941966; doi:10.1186/s13104-021-05503-0)
Supplement: Supplementary file 2 — Additional file 2: Frequency and Percent of bacterial isolates recovered form hospitalized patients. [file 13104_2021_5503_MOESM2_ESM.docx]

**Additional file 2: Frequency and Percent of bacterial isolates recovered form hospitalized patients**

| Bacterial Isolates | Frequency | Percent |
| --- | --- | --- |
| *Escherichia coil* | 360 | 70.7% |
| *Citrobacter* | 25 | 4.9% |
| *Klebsiella* | 44 | 8.6% |
| *Enterobacter* | 16 | 3.1% |
| *Serratia* | 1 | 0.2% |
| *Proteus* | 2 | 0.4% |
| *Pseudomonas* | 9 | 1.8%% |
| *Acinetobacter* | 3 | 0.6% |
| *Staphylococcus aureus* | 23 | 4.5% |
| *Staphylococcus saprophyticus* | 10 | 2.0% |
| *Staphylococcus epidermidis* | 8 | 1.6% |
| *Enterococcus faecalis* | 4 | 0.8% |
| *Streptococcus agalactiae* | 3 | 0.6% |
| *Shigella sonnei* | 1 | 0.2% |
| Total | 509 | 100.0 |
