# Supplementary material for: A retrospective cross-sectional survey on nosocomial bacterial infections and their antimicrobial susceptibility patterns in hospitalized patients in northwest of Iran
Source: BMC Res Notes. 2021 Mar 9;14:88. doi: 10.1186/s13104-021-05503-0 (PMC7941966; doi:10.1186/s13104-021-05503-0)
Supplement: Supplementary file 3 — Additional file 3: Frequency of multi and extensively- drug resistant (MDR and XDR) Gram-negative isolated bacteria (n %) [file 13104_2021_5503_MOESM3_ESM.docx]

**Additional file 3**: Frequency of multi and extensively- drug resistant (MDR and XDR) gram-negative isolated bacteria

| Bacterial Isolates | MDR | XDR |
| --- | --- | --- |
| *Escherichia coli* | 274 (76.1 %) | 188 (52.2%) |
| *Citribacter spp* | 24 (96.0%) | 17 (68.0%) |
| *Klebsiella* *spp* | 41(93.1%) | 35 (79.5%) |
| *Enterobacter* *spp* | 16 (100.0%) | 12 (75.0%) |
| *Serratia* | 1 (100.0%) | 0 (0)% |
| *Proteus spp* | 1 (100.0%) | 0 (0%) |
| *Pseudomonas aeruginosa* | 9 (100.0%) | 8 (88.9%) |
| *Acinetobacter* | 3 (100.0%) | 3 (100%) |
| *Shigella sonnei* | 1 (100.0%) | 1 (100.0%) |
| Total | 370 (80.5%) | 264 (57.3%) |
