# Supplementary material for: A retrospective cross-sectional survey on nosocomial bacterial infections and their antimicrobial susceptibility patterns in hospitalized patients in northwest of Iran
Source: BMC Res Notes. 2021 Mar 9;14:88. doi: 10.1186/s13104-021-05503-0 (PMC7941966; doi:10.1186/s13104-021-05503-0)
Supplement: Supplementary file 4 — Additional file 4: Frequency of MDR and XDR Gram positive bacterial isolates (n %). [file 13104_2021_5503_MOESM4_ESM.docx]

**Additional file 4:** frequency of MDR and XDR gram positive bacterial isolates ( n%).

| **Bacterial Isolates** | **MDR** | **XDR** |
| --- | --- | --- |
| ***Staphylococcus aureus*** | 22 (95.7%) | 20 (87.0%) |
| ***Staphylococcus saprophyticus*** | 9 (90.0%) | 4 (40.0%) |
| ***Staphylococcus epidermidis*** | 7 (87.5%) | 6 (75.0%) |
| ***Enterococcus faecalis*** | 4 (100.0%) | 4 (100.0%) |
| ***Streptococcus agalactiae*** | 2 (66.6%) | 1 (33.3%) |
| **Total** | 44 (91.7%) | 35 (72.9%) |
